# A Rare Case of Left Ventricular Malignant Peripheral Nerve Sheath Tumour—Case Report and Review of the Literature

**DOI:** 10.3390/medicina58101404

**Published:** 2022-10-06

**Authors:** Horațiu Moldovan, Irina Ciomaga, Elena Nechifor, Robert Tigănașu, Aida Badea, Irina Dobra, Claudia Nica, Costin Scarlat, Daniela Gheorghiță, Iulian Antoniac, Ondin Zaharia

**Affiliations:** 1 Faculty of Medicine, Carol Davila University of Medicine and Pharmacy, 050474 Bucharest, Romania; 2 Department of Cardiovascular Surgery, Emergency Clinical Hospital Bucharest, 014461 Bucharest, Romania; 3 Academy of Romanian Scientists, 54, Spl. Independentei, 050711 Bucharest, Romania; 4 Department of Cardiovascular Surgery, Sanador Clinical Hospital, 010991 Bucharest, Romania; 5 Faculty of Materials Science and Engineering, Politehnica University of Bucharest, 060042 Bucharest, Romania; 6 Department of Internal Medicine, Prof. Dr. Theodor Burghele Clinical Hospital, 050659 Bucharest, Romania

**Keywords:** soft tissue sarcoma, malignant primary cardiac tumour

## Abstract

Malignant peripheral nerve sheath tumour (MPNST) is an aggressive and uncommon cancer developing in the peripheral nerve sheath. Primary cardiac MPNST is an extremely rare finding, with no specific imaging and clinical characteristics. Only a handful of cases have been reported in the literature; thus, little is still known about them. Cardiac computed tomography (CT) and cardiac magnetic resonance imaging (CMRI) are important means of assessing cardiac morphology and function. The preferred course of treatment for this pathology is by full surgical resection of the tumour, with negative (clear) margins, followed by adjuvant radiotherapy and chemotherapy. We present the case of a 42-year-old woman with no significant cardiovascular symptoms who was incidentally diagnosed during routine transthoracic echocardiography (TTE) with a cardiac mass located in the left ventricle.

## 1. Introduction

Malignant peripheral nerve sheath tumour (MPNST) is a rare type of soft tissue sarcoma, found in approximately 2% of all sarcomas, with a high rate of recurrence and a high rate of metastatic dissemination. Cardiac primary malignancies are rare, with an incidence ranging from 0.001 to 0.03%. Amongst all phenotypes of malignant primary cardiac tumours, MPNSTs are exceedingly rare, with an incidence of 0.75% in all primary cardiac tumours [1,2,3]. Due to their rarity, little is known about their specific clinical manifestations, imaging features and proper management, though the preferred course of treatment seems to be through complete surgical resection, with negative margins, followed by adjuvant radiotherapy and chemotherapy to lower the risk of recurrence and metastatic dissemination [4,5]. Cardiac computed tomography (CT) and cardiac magnetic resonance imaging (CMRI) are important means of assessing cardiac morphology and function. TTE is a fast, non-invasive method for demonstrating the presence and location of a cardiac mass. The diagnosis and classification of MPNST is a challenge because these types of tumours are difficult to differentiate from neurofibroma; these types of tumours often take a similar form to synovial sarcoma and ossifying fibromyxoid tumours. Therefore, these types of borderline tumours are difficult to diagnose, and this represents a problem in the establishment of therapeutic conduits, such as surgical treatment [6].

According to the genetic studies, mutations and their incidence rate were identified at the level of the following genes: NF1 (56/64 = 87.5%). SUZ12 (69/123 = 56.1%), EED (40/123 = 32.5%), TP53 (29/72 = 40.3%), and CDKN2A (54/72 = 75.0%), which led to an increased understanding of the complexity of the MPNST genome [7].

## 2. Case Report

**Figure 1:**
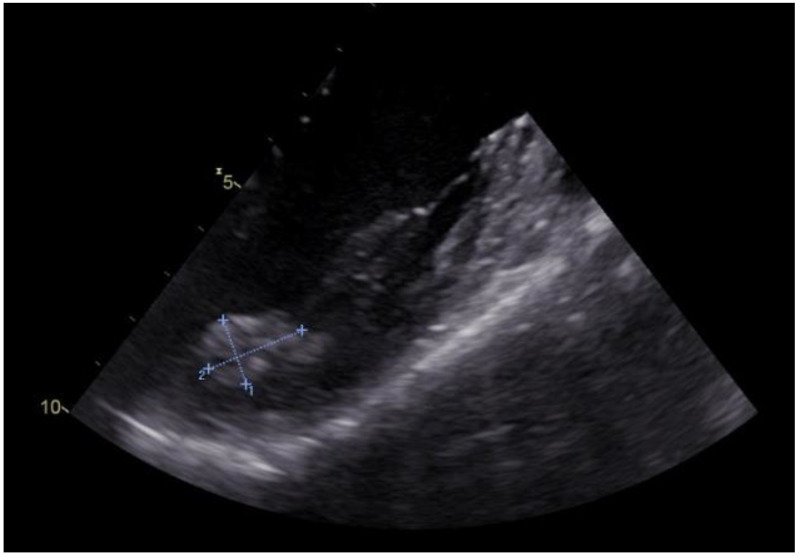
Two-dimensional transesophageal echocardiography of the tumour in the left ventricle and papillary muscle at the base of the anterolateral papillary muscle (orange arrow, tumour; blue arrow, anterolateral papillary muscle).

We present the case of a 42-year-old woman with no significant cardiovascular symptoms admitted to the hospital for further medical investigations after the incidental discovery of a left ventricular mass during a routine TTE presented in Figure 1.

The patient had a personal history of euthyroid autoimmune thyroiditis and minor beta-thalassemia and no history of surgical interventions. Worth mentioning in her family history is her father’s pulmonary neoplasm, diagnosed at the age of 57. The TTE at admission revealed no valvular dysfunction, a left ventricle (LV) with normal systolic function, with a left ventricle ejection fraction (LVEF) of 60%, but the presence of a hyperechogenic, homogenous mass in the LV, at the base of the anterolateral papillary muscle, apparently without myocardial invasion, immobile, of approximately 23/11 mm.

**Figure 2:**
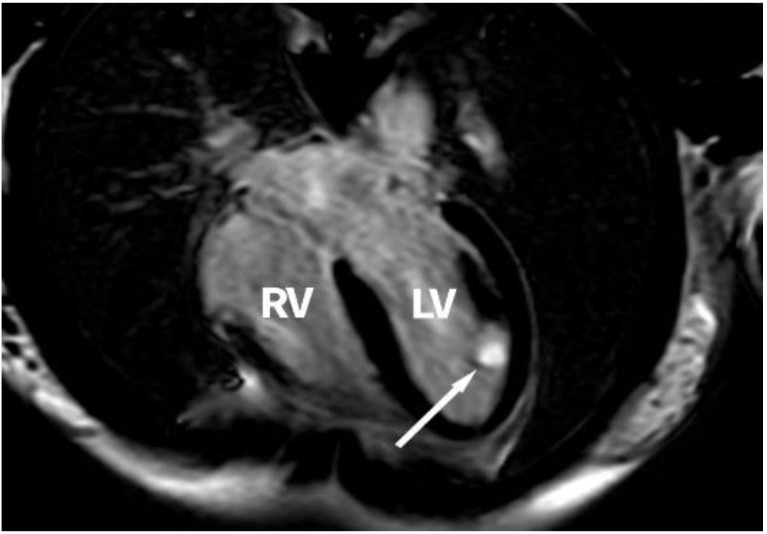
Cardiac magnetic resonance image of the tumour in the left ventricle and papillary muscle at the base of the anterolateral papillary muscle (white arrow, left ventricle tumour); RV, right ventricle; LV, left ventricle.

The CMRI performed showed a tumour mass of 18/14/12 mm located inside the LV, at the base of the anterior papillary muscle, in contact with the lateral wall, with well-defined margins, partially mobile, with no evidence of an intraventricular obstructive effect, with homogenous T1 and T2 isointense signals, on the myocardial perfusion sequence with a lower perfusion rate than that of the myocardium, with late oncoming, intense and homogenous enhancement after the management of contrast agent on the LGE sequences, the tumour mass not presenting malignancy characteristics. Figure 2 presents a cardiac magnetic resonance image of the tumour.

**Figure 3:**
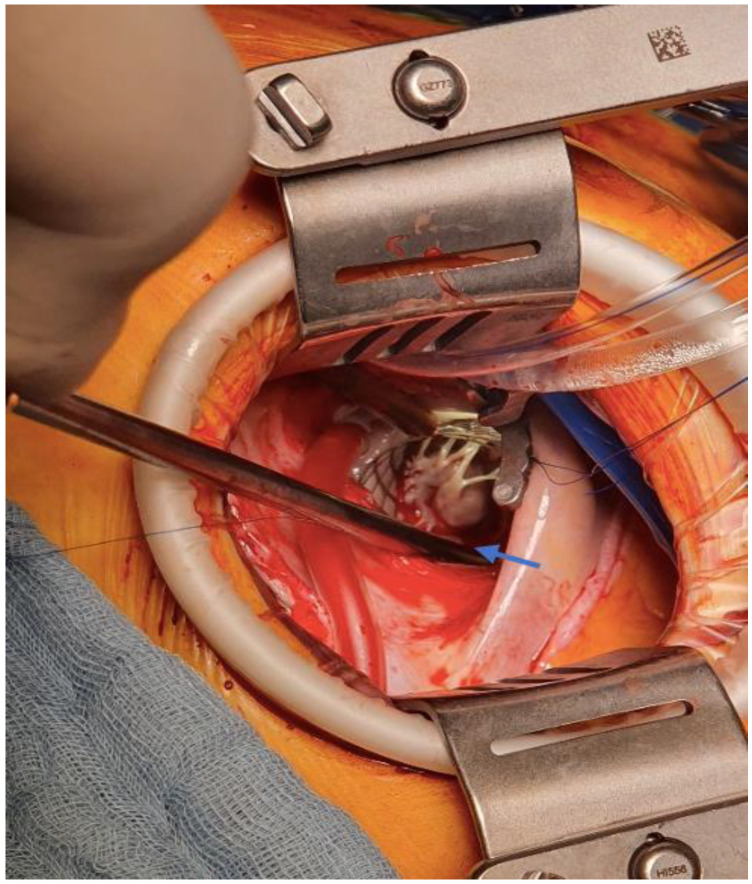
Left ventricle tumour implanted at the base of the anterolateral papillary muscle (blue arrow).

**Figure 4:**
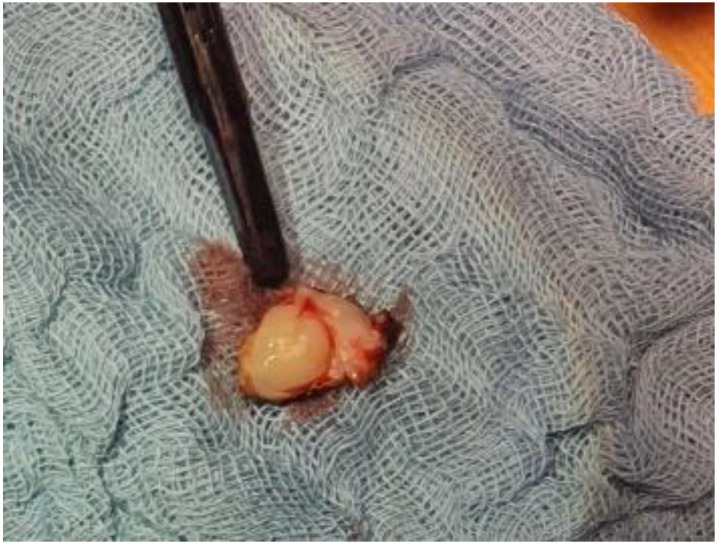
Intraoperative image of the excised tumour.

The patient was then referred to our service with the indication of surgical removal of the left ventricular tumour mass. The approach was through a video-assisted thoracoscopic with anterolateral mini-thoracotomy under general anaesthesia, followed by peripheral cannulation with cardiopulmonary bypass, aortic cross-clamp, and aortic antegrade cardioplegia administration. A left atriotomy was performed at the level of the interatrial groove. The pearlescent white left ventricular tumour mass was seen through the mitral valve, implanted at the base of the anterolateral papillary muscle, immobile and is presented in Figure 3. The mass was excised without damaging the anterolateral papillary muscle. Figure 4 presents the excised tumour.

The left atrium was then closed in the usual fashion. The aorta was unclamped, and then the patient was slowly weaned off cardiopulmonary bypass. The intraoperative transesophageal echocardiography revealed mild mitral valve regurgitation. The excised tumour was sent for histological and immunohistochemical examination.

The postoperative evolution was uneventful, and the patient was extubated on postoperative day 1, and there was no need for vasopressors or inotropes, the only notable events being several short episodes of atrial fibrillation pharmacologically converted to sinus rhythm using amiodarone.

**Figure 5:**
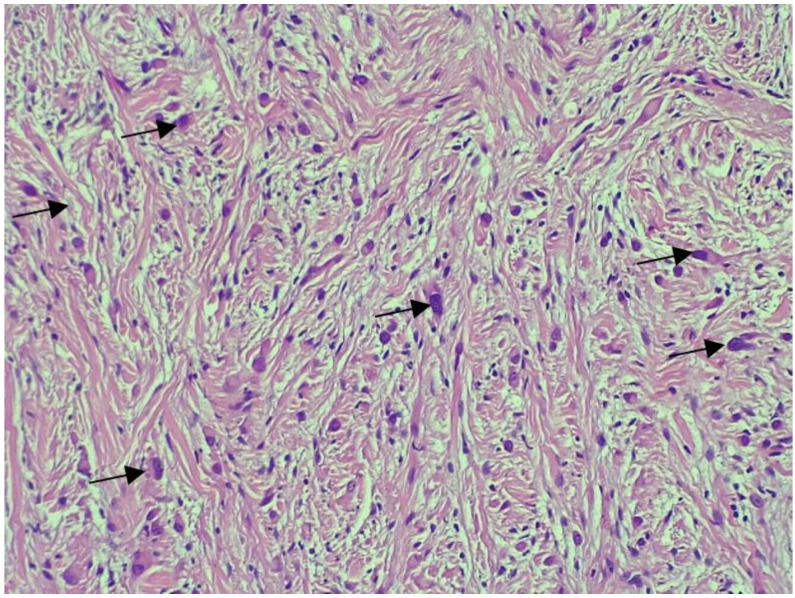
Tumoural proliferation with fusiform cells and myxoid stroma that infiltrate the myocardial fibres and mild degree of nuclear atypia.

**Figure 6:**
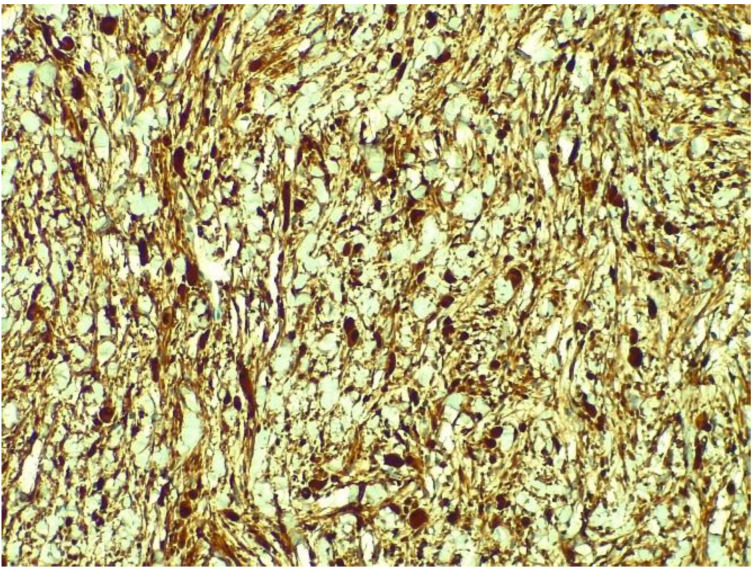
Immunohistochemical examination showing S100 marker diffusely positive in the fusiform cells.

**Figure 7:**
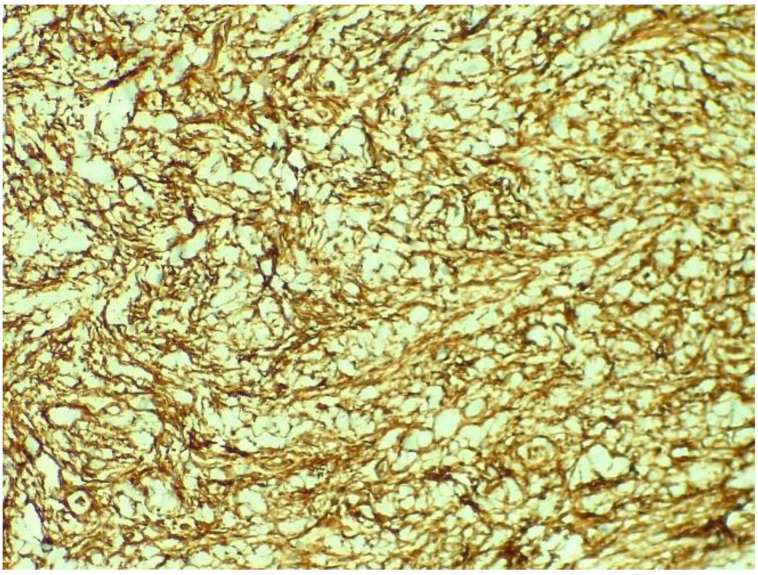
Immunohistochemical examination showing CD34 marker positive in the vessels and the fusiform cells.

Figure 5 presents the histopathological examination of the tumour revealed tumoural proliferation with fusiform cells and myxoid stroma that infiltrate the myocardial fibres, a mild degree of nuclear atypia, no visible mitosis and no necrosis. The immunohistochemical examination showed the S100 marker diffusely positive in the fusiform cells, as presented in Figure 6, positive actin marker in the vessels and CD34 marker positive in the vessels and the fusiform cells, as presented in Figure 7. A diagnosis of low-grade malignant peripheral nerve sheath tumour was then confirmed. The patient was referred to an oncologist for further examinations and treatment options.

The patient was discharged 7 days after surgery. Discharge echocardiography showed no notable changes in the left and right ventricular functions, an LVEF of 60%, and mild mitral and tricuspid regurgitations. 

## 3. Discussions

MPNST is a rare type of soft tissue sarcoma. It primarily affects the extremities, but it can also rarely originate from the heart. Most of the MPNSTs that affect the heart occur on the right side, close to the interatrial septum. The reason for this location might be the origin of the MPNST from the cardiac branches of the vagus nerve [8,9,10,11]. A few cases have been reported on the left side of the heart. About 50% of these tumours arise de novo, the other half arise from neurofibromas in the context of type 1 neurofibromatosis [9,10,11,12,13,14,15,16,17,18,19]. Patients with neurofibromatosis type I have an 8–13% greater risk of developing an MPNST than the general population [19,20]. Our patient had no features of neurofibromatosis type I. She had no cardiovascular symptoms, her intracardiac tumour being fortunately diagnosed after a routine TTE. Echocardiography is a fast, non-invasive, low-cost imaging tool that provides the first idea of the tumour’s aetiology and is able to obtain information about the location, mobility, appearance and attachment of the tumour that can help determine if the mass is benign or malignant. Most of the time, TTE is performed for another indication and may be the first imaging tool that determines the presence of a cardiac mass [21]. Even though there is always the need for histopathological confirmation of the diagnosis, some imaging features and aiding tools, such as contrast imaging, can help distinguish between the different types of cardiac masses [22]. Therefore, the question arises of whether to start a screening program using TTE to determine the presence of intracardiac masses. Knowing the low incidence rate of intracardiac tumours and the widespread prevalence, with masses appearing in the paediatric population as well as in the young adult population and in the elderly, with no significant evidence of increased prevalence in a specific age group, we consider that it would not be cost-effective to start a screening program using TTE for discovering intracardiac tumours [23].

MPNST is an aggressive high-grade tumour with a high mitotic rate for which an accurate histological diagnosis is made difficult by the lack of specific histological and immunohistochemical markers and by the lack of standardised diagnostic criteria [3,4,15,17,24,25]. That being said, S-protein is found in 50–90% of patients, which was also seen in our case, Leu-7 marker is found in approximately 50%, Ki-67 in 5–65% of patients and myelin basic protein in 40% [12,14,18,26]. CMRI can accurately locate the tumour and asses the extension and invasion in the surrounding tissue. It can also reveal some features specific to a malignant tumour, such as perilesional oedema, contrast enhancement and irregular margins [27]. However, a precise diagnosis cannot be formulated on imaging findings alone due to the limited cases reported. In a retrospective study, Yun and Winfree [28] demonstrated that by using a classification system based on imaging findings and clinical features, they managed to obtain perfect performance, with 100% specificity and sensitivity in diagnosing MPNSTs. Thus, it is important to combine all of the factors, the clinical presentation and the radiological and histological findings in trying to sustain a more precise diagnosis.

A case should be made for the use of endomyocardial biopsies (EMB) for the histological diagnosis of intracardiac masses, even though clinical diagnosis could mainly be performed using imaging tools. The value of EMBs rests in the possibility of providing critical information for treatment and prognosis. The main indications for EMBs in cardiac masses are for investigating right heart tumours with different types of growth patterns, such as infiltrative or obstructive, and for the differential diagnosis of masses with malignancy features. Furthermore, left-sided EMBs have been shown to be a safe and feasible method for investigating the left ventricle endomyocardium [29]. There are different modalities for guiding the EMBs, such as through 2D or 3D transoesophageal echocardiography (TEE), fluoroscopic guidance or intracardiac echocardiography (ICE). Out of these three options, the preferred method seems to be ICE guidance, which offers the direct visualisation of the mass and can be performed with conscious sedation, thus minimising the risk of perforation. TEE requires general anaesthesia and an experienced echocardiographist, while the fluoroscopic guiding drawbacks consist of lower accuracy and longer exposure to radiation [30]. 

The preferred and most effective course of treatment for MPNSTs is through complete surgical excision, even though complete resection is not always possible. The overall 5-year survival rate for MPNSTs is approximately 44–50%, depending on different factors concerning the patient’s age, tumour location and the size of the mass. Larger masses tend to have a poorer prognosis, making this factor the most valid independent prognostic factor for the 5-year survival rate of patients with MPNST [8,10,11,12,13,14,19,26]. Adjuvant radiotherapy can be used, though radiotherapy and chemotherapy tend to have little effect on the patient’s survival. Higher survival rates have been seen in cases with small tumour sizes (<5 cm), complete excision and neoadjuvant therapy, early diagnosis and low-grade characteristics of the tumour [28,31,32,33]. Regarding the surgical approach, intracardiac masses have been traditionally treated through median sternotomy for better exposure and the larger surgical field. However, with the development of minimally invasive techniques, a new solution emerged for treating small tumours. A minimally invasive approach reduces blood loss, shortens the hospitalisation period, and is associated with improved healing time and with better cosmetic results [34].

Shuang Li et al. [26] managed to gather from the literature eight cases of patients with MPNSTs, five males and three females, originating from the heart chambers, pericardium, myocardium or the root of the great vessels. The mean age of diagnosis was 33.5 years. The most frequent clinical manifestation was shortness of breath, occurring in five out of eight cases discussed in the study. Other manifestations were nonspecific, including chest pain, weight loss or oedema, indicating that the clinical manifestations of cardiac MPNSTs depend on the size and location of the tumour. Our patient presented no clinical manifestations, her cardiac tumour being discovered at a routine TTE. Out of the eight cases, only one tumour originated in the left ventricle, and four were located in the atria (two left, two right), two in the pericardium and one was on the mitral valve. For the eight cases reported, the tumour was discovered using TTE. Six patients were further examined through CT and two using CMRI. TTE was useful in showing the presence and position of the masses, the CT examination revealed more details, but CMRI pinpointed the exact location of the tumour, managing also to assess the extension of the mass and its invasion of the neighbouring structures. Whilst reviewing the current literature on the subject, CMRI has been proven to be the most precise mean of evaluating surgical resectability due to its high tissue resolution (increased sensibility and specificity). Only five cases underwent total surgical excision of the mass, four of whom had a short-term favourable outcome. Adjuvant chemotherapy and radiotherapy were used in only two patients, making it difficult to assess their effectiveness [26,35].

Almost half of the cases associate NF1 with a gene mutation on chromosome 17. Due to the variability of the nomenclature in the past and the different diagnostic criteria, very few cases have been reported. Several cases of intracardiac MPNST were cited in PubMed, which have been confirmed by histopathological examination. In this study, seven patients were included, five men and two women, the most relevant studies were focused on clinical manifestations. Following this study, it was established that there is no gender or age predilection for this type of tumour. The most common clinical manifestation was dyspnea (25%), followed by other nonspecific manifestations, such as oedema, retrosternal pain, fatigue, and weight loss, all these symptoms being influenced by the location and size of the tumour. Lesions were studied, coming from two right atria, two left atria, one from the left ventricle, mitral valve, and two in the pericardium [8,36]. The most common imaging methods for diagnosing this formation are TTE, MRI and CT.

## 4. Conclusions

MPNST is a rare type of soft tissue sarcoma with nonspecific clinical and imaging characteristics. Most often, the tumours are discovered through TTE, being able to indicate their presence and position. Details about the exact location, extent and invasion of the tumour in the neighbouring tissues can only be obtained through CMRI, thus being considered the main modality of evaluating the surgical resectability of the masses. A case should be made for the use of EMBs for the histological diagnosis of intracardiac masses, having the possibility of providing critical information about treatment and prognosis. To sustain a more precise diagnosis and in order to better assess the best course of treatment, it is important to combine all factors, the clinical presentation, the radiological and histological findings. The preferred and most effective course of treatment for MPNSTs is through complete surgical excision, even though complete resection is not always possible. Adjuvant radiotherapy can be used, though radiotherapy and chemotherapy tend to have little effect on the patient’s survival rate.

Differential diagnosis of MPNST can be difficult due to its morphological variability. The problems in the diagnosis of these tumours arise because of the very few differences between benign tumours and MPNST. Because of the available molecular techniques, molecular diagnosis seems to have an important role shortly. For the accuracy of the diagnosis, good management, and remote follow-up of the patient, it is necessary to create an efficient collaboration of a multidisciplinary team.

A combination of clinical, immunohistochemistry and pathological helps in diagnosing these tumours. The overall treatment approach should be similar to that of any other high-grade sarcomas. Postoperative radiotherapy has a definite role in both disease-free and overall survival. Given the rarity of this entity and conflicting reports, it is difficult to define the role of radiation in the management of MPNSTs. Currently, postoperative radiotherapy is recommended by the oncology consensus group as part of a uniform treatment policy for MPNSTs, similar to other high-grade soft tissue sarcomas, despite having clear surgical margins.

## Data Availability

The data presented in this study are available on reasonable request from the corresponding author.
